# Vitamin D Alleviates Heavy Metal-Induced Cytotoxic Effects on Human Bone Osteoblasts Via the Induction of Bioenergetic Disruption, Oxidative Stress, and Apoptosis

**DOI:** 10.1007/s12011-024-04337-8

**Published:** 2024-09-05

**Authors:** Ekramy M. Elmorsy, Ayat B. Al-Ghafari, Huda A. Al Doghaither, Majed Gorayan Alrowaili, Zenat Ahmed Khired, Eman A. Toraih, Manal S. Fawzy, Shaimaa A. Shehata

**Affiliations:** 1 Pathology Department, Faculty of Medicine, Northern Border University, 91431 Arar, Saudi Arabia; 2 Center for Health Research, Northern Border University, Arar, Saudi Arabia; 3 Department of Biochemistry, Faculty of Science, King Abdulaziz University, 21589 Jeddah, Saudi Arabia; 4 Experimental Biochemistry Unit, King Fahd Medical Research Center, King Abdulaziz University, 21589 Jeddah, Saudi Arabia; 5 Department of Surgery (Orthopedic Division), Faculty of Medicine, Northern Border University, Arar, Saudi Arabia; 6 Department of Surgery, College of Medicine, Jazan University, 45142 Jazan, Saudi Arabia; 7 Department of Surgery, School of Medicine, Tulane University, New Orleans, LA 70112 USA; 8 Genetics Unit, Department of Histology and Cell Biology, Suez Canal University, Ismailia, 41522 Egypt; 9 Department of Biochemistry, Faculty of Medicine, Northern Border University, 73213 Arar, Saudi Arabia; 10 Department of Medical Biochemistry and Molecular Biology, Faculty of Medicine, Suez Canal University, Ismailia, 41522 Egypt; 11 Department of Forensic Medicine and Clinical Toxicology, Faculty of Medicine, Suez Canal University, Ismailia, 41522 Egypt

**Keywords:** Antioxidants, Bone, Cadmium, Lead, Osteoblasts, Redox stress, Vitamin D

## Abstract

Cadmium (Cd) and lead (Pb) are heavy metals (HMs) that persistently contaminate the ecosystem, and bioaccumulation in bones is a health concern. We used biochemical and molecular assays to assess the cytoprotective effect of vitamin D (VD) on Cd- and Pd-induced chemical toxicity of human bone osteoblasts in vitro. Exposing Cd and Pb to human osteoblast cultures at concentrations of 0.1–1000 µM for 24–72 h significantly reduced osteoblast viability in an exposure time- and concentration-dependent manner. The cytotoxic effect of Cd on osteoblasts was more severe than Pb’s, with 72-h exposure estimated half maximal effective concentration (EC50) of 8 and 12 µM, respectively, and VD (1 and 10 nM) alleviated cytotoxicity. Bioenergetics assays of ATP, mitochondrial membrane potential, and mitochondrial complex I and III activity showed that both Cd and Pb (1 and 10 µM) inhibited cellular bioenergetics after 72-h exposure. Cd and Pb increased lipid peroxidation and reactive oxygen species with reduced catalase/superoxide dismutase antioxidant activities and increased activity of caspases -3, -8, and -9. Co-treatment with VD (1 and 10 nM) counteracted bioenergetic disruption, oxidative damage, and apoptosis in a concentration-dependent manner. These findings suggest that VD is effective in managing the toxic effects of environmental pollutants and in treating bone diseases characterized by oxidative stress, apoptosis, and bioenergetic disruption.

**Graphical Abstract:**

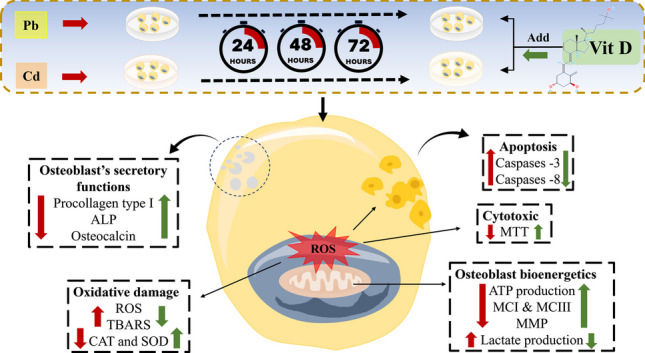

## Introduction

Heavy metals (HMs), including cadmium (Cd), lead (Pb), arsenic (As), and mercury (Hg), are global environmental pollutants that have detrimental and often disastrous effects on human health [1]. For certain HMs, bone is a primary site of accumulation and toxic effects; for example, Pb can be stored and sequestered in bones and can later remobilize with neurotoxic or teratogenic effects during childhood or pregnancy [2]. Pb exposure in early childhood has been linked to higher bone mineral density (BMD) [3]; however, in older children, adolescents, and adults, Pb has been associated with lower BMD [4, 5].

Environmental Cd poisoning is usually characterized by kidney damage, but exposure can also be harmful to bone. Clinical outcomes for humans exposed to environmental Cd include reduced bone density (osteoporosis) and softening of the bones (osteomalacia), both of which increase the risk of fractures. Cd exposure can also cause renal tubular osteomalacia (Itai-Itai disease), characterized by renal insufficiency and painful bones secondary to osteomalacia. [6–8].

Several organs are susceptible to damage from HMs, but exposure can also have secondary effects on metabolism that affect calcium hemostasis and VD activation [9–11]. HMs may also directly affect bone cells, impairing resorption or creating new bone [11].

The bone structure comprises 10% cells, 30% organic matrix, and 60% mineral crystals. There are four types of bone cells: osteocytes, osteoclasts, osteoblasts, and bone-lining cells. Osteoblasts, differentiated cuboidal cells that cover the bone surface, are responsible for type I collagen and non-collagenous protein secretions, such as alkaline phosphatase (ALP), osteopontin, osteocalcin, and bone sialoprotein II, all of which are necessary for bone mineralization [12].

The active type of vitamin D3 (VD) in humans is l, 25-dihydroxy cholecalciferol (l, 25-[OH]_2_D3), which is produced in the liver via 25-hydroxylase and in the kidneys via 25-hydroxyvitamin D-1 alpha-hydroxylase (also called Cyp27b1). [13]. Low blood Ca^2+^ levels return to normal when VD binds to the VD receptor (VDR), which induces calmodulin and other calcium-binding proteins that facilitate Ca^2+^ absorption. When adequate serum calcium is present, active VD, a fat-soluble vitamin, facilitates bone mineralization [14]. It ameliorates bone metabolism illnesses, including multiple osteosclerosis, osteoporosis, osteomalacia, rheumatoid arthritis, and tibial dyschondroplasia [15].

VD and its metabolites are crucial to the production of human bone marrow stromal cells (hBMSCs) and the development of osteoblasts by upregulating collagen I expression, osteocalcin (OCN) synthesis, ALP activity, and mRNA levels [16]. Also, the active form of VD protects nucleus pulposus-derived mesenchymal stem cells (NPMSCs) from hydrogen peroxide (H_2_O_2_)-induced apoptosis by reducing apoptosis rate and the expression of apoptosis-related proteins like cleaved caspase-3, Bcl-2, and Bcl-2 associated X-protein (Bax) [17]. Understanding the protective effects of various agents and chemicals against heavy metal-induced toxic effects on bones is crucial to global human health. In this sense, the present study assesses the protective effect of VD against Cd and Pb-induced bone toxicity in human osteoblasts.

## Material and Methods

### Chemicals and Reagents

We used the HM solutions cadmium dichloride and lead nitrate. All associated chemicals were obtained from Sigma (St. Louis, MO, USA) unless stated otherwise.

### Cell Culture

For all steps in the current study, osteoblasts in 96-well plates were used with 4–5 passages. Cells were cultured in human osteoblast growth medium and incubated at 37 °C, 5% CO_2_ in a humidified incubator.

### Cytotoxicity Assessment Using Methyl Tetrazolium (MTT) and Lactate Dehydrogenase Leakage (LDH) Assays

Osteoblasts were grown to 90% confluence after being seeded at a density of 2 × 10^4^ cells/well, then exposed to cadmium dichloride (Cd) or lead nitrate (Pb) for 24, 48, or 72 h at doses of 0.1, 1, 10, 100, and 1000 µM as detailed below. Cell viability and metabolic activity were assessed by measuring the generation of reduced MTT substrate at 590 nm via spectrophotometry based on the manufacturer’s protocol. Every assay point was carried out three times. The values of MTT absorbance of different samples exposed to HMs were reported as percentages with an absorbance reference control (100%). The MTT assay was remeasured after adding VD. Osteoblasts were cotreated with vitamin D (1 or 10 nM), and both HMs were assayed at doses of 1 and 10 µM for 72 h.

### Effect of HMs on Osteocalcin Secretion

In 96-well plates, osteoblasts were seeded at a density of 4 × 10^3^ cells/well and cultivated to 80–90% confluency. Cells received a 72-h co-treatment of Pb or Cd (1 µM or 10 µM) and VD (1 nM or 10 nM). Media were gathered using a sandwich enzyme-linked immunosorbent assay (ELISA) kit (Takara Shuzou, Japan), and osteocalcin levels were calculated by extrapolating osteocalcin concentrations from a standard curve.

### Effect of HMs on Alkaline Phosphatase (ALP) Secretion

In 24-well plates, osteoblasts were seeded at a density of 4 × 10^6^ cells/well and cultivated to 80–90% confluency. Cells that underwent treatment received a 72-h co-treatment of Pb or Cd (1 or 10 µM) and VD (1 or 10 nM). ALP activity in the media was measured using a commercial p-nitrophenyl phosphatase assay per the manufacturer’s instructions (Abcam, Cambridge, UK). ALP activity assessment results were quantified by spectrophotometry using a TopCount plate reader (Perkin Elmer, Ueberlingen, Germany) at 405 nm.

### Effect of HMs on Procollagen Secretion

In 96-well plates, osteoblasts were seeded at a density of 4 × 10^3^ cells/well and cultivated to 80–90% confluency. Cells then received a 72-h co-treatment of Pb or Cd (1 or 10 µM) and VD (1 or 10 nM) to measure procollagen type I C-peptide (PIP). Cell homogenization was performed using phosphate-buffered saline containing 1 mM ethylenediaminetetraacetic acid, 0.5% Triton X-100, and 1 mM phenylmethylsulfonyl fluoride. PIP was assessed using an ELISA kit (Takara Shuzou, Japan). PIP concentrations were extrapolated from a standard curve. Procollagen content was calculated as medium and normalized to cell number.

### Measurements of Intracellular Adenosine Triphosphate (ATP) Levels

Before being treated, osteoblasts were sown at a density of 2 × 10^4^ cells/well and cultivated to 90% confluence. Cells then received a 72-h co-treatment of Pb or Cd (1 or 10 µM) and VD (1 or 10 nM), and intracellular ATP concentrations were determined (Abcam, Cambridge, UK). Single photon counting was used to detect ATP concentrations, which are expressed as luminescence values that were assessed concerning readings from control wells.

### Measurements of Mitochondrial Membrane Potential (MMP)

In 24-well plates, osteoblasts were seeded at a density of 3 × 10^4^ cells/well and exposed to Pb or Cd (1 or 10 µM), and VD (1 or 10 nM) was added as a co-treatment. After 72 h, the media were eliminated, and the cells were incubated at 37 °C with a MitoTracker Green staining solution (50 nM). Stained cells were kept in new phosphate-buffered saline every 30 min. A TopCount fluorescent microplate reader (Perkin Elmer, Ueberlingen, Germany) was used to measure the fluorescence at 490 nm and 516 nm emission and excitation filters, respectively. To cause membrane uncoupling, carbonyl cyanide-4-(trifluoromethoxy)phenylhydrazone (FCCP) was employed as a positive control.

### Measurements of Mitochondrial Complex (MC) I and III Activity

To determine how vitamin D affects osteoblasts MC I and III, Pb or Cd (1 and 10 µM) and VD (1 or 10 nM) were co-incubated with cells for 72 h. The mitochondrial enriched fraction of cells (for the complex I assay) and the isolated cell lysate (for the complex III assays) were prepared using the procedure outlined by [18]. For the complex I assay, the mitochondrial enriched fraction was subjected to three freeze–thaw cycles in liquid nitrogen before use to cause damage to mitochondrial membranes. Rotenone was used as an MCI selective inhibitor, while antimycin A was used as an MCII inhibitor to standardize complex activities. Assays were performed at least five times to ensure robust data.

### Measurements of Lactate Production

In each well of a 24-well plate, 10^4^ cells were sown. Cells received a 72-h co-treatment of Pb or Cd (1 or 10 µM) and VD (1 or 10 nM). After trypsinization, the cells were centrifuged for 5 min at 1000 × g. Pelleted cells were counted, and the supernatant medium was kept. Lactate production readings were calculated using a lactate assay following the manufacturer’s instructions (Biovision, Mountain View, California, USA). Lactate production was expressed as a proportion of control lactate production after being standardized to cell number. This procedure was performed five times.

### Measurements of Reactive Oxygen Species (ROS)

We determined the amount of ROS produced after 72-h co-treatment with Pb or Cd (1 or 10 µM) and VD (1 or 10 nM) using a 2,7-dichlorodihydrofluorescein diacetate (DCFDA) assay as previously described [19]. Osteoblasts treated with antimycin A (10 mM) for 30 min served as a positive control, and non-stained cells provided negative control data.

### Measurements of Superoxide Dismutase (SOD) and Catalase (CAT) Activity

To determine the effects of HMs and VD on the activity of antioxidant enzymes, superoxide dismutase (SOD) and catalase (CAT) from osteoblasts were assessed after treatment with Pb, or Cd (1 or 10 µM) and VD (1 or 10 nM) for 72 h. CAT activity was measured calorimetrically as described previously [20]. After cell harvesting, each sample’s protein content was determined, and the tissue homogenate (0.1 ml) was mixed with 1 ml of 0.01 M pH 7 phosphate buffer and 0.4 ml of 2 M H_2_O_2_, followed by a 2 ml dose of dichromate-acetic acid reagent to halt the reaction. At 620 nm, absorbance was measured. The unit of measurement for CAT activity was µmoles H_2_O_2_ consumed/min/mg of protein.

A colorimetric commercial assay kit (Abcam, Cambridge, UK) was used to measure SOD activity following the instructions provided. Cells were taken out, lysed, and centrifuged at 4 °C for 5 min at 14,000 × g. After collection, the supernatants were frozen until analysis. Both cytosolic and mitochondrial SOD were present in the supernatant. Using a TopCount plate reader (Perkin Elmer, Ueberlingen, Germany), absorbance was measured at 440 nm. Plotting against a standard curve was used to measure SOD activity units, and results were reported in milligrams of total protein. Each experiment was performed in triplicate.

### Measurements of Lipid Peroxidation

Lipid peroxidation is commonly detected using thiobarbituric acid reactive substances (TBARS). After treating osteoblasts with Pb or Cd (1 or 10 µM) and VD (1 or 10 nM) for 72 h, TBARS was measured using a commercial kit (Abcam) that measured malondialdehyde (MDA), a significant lipid oxidation product. Cells were taken, and a Dounce homogenizer on ice was used to homogenize them using the MDA lysis buffer. After sonicating the lysate and centrifuging it at 13,000 × g, the supernatant was separated and saved for further examination. A TopCount plate reader (Perkin Elmer, Ueberlingen, Germany) was used to detect absorbance at 532 nm.

### Measurement of Caspases -3, -8, and -9 Activities

BD Apo-Alert caspase fluorescence assay kits (Clontech Laboratories, Palo Alto, CA) were used to determine the effect of HMs, in the presence and absence of VD, on osteoblast caspases 3 (common apoptosis pathway), 9 (intrinsic apoptosis pathway), and 8 (extrinsic apoptotic pathway) activities following the provided instructions. For 72 h, cells were exposed to Pb or Cd (1 or 10 µM) and VD (1 or 10 nM). For the complex 3, 8, and 9 assays, fluorescence was measured in 96-well plates using the Synergy HT Fluoremeter plate reader (Bio-tek Instruments, Inc., Winooski, VT) at excitation/emission wavelengths of 400/505, 400/505, and 380/460 nm, respectively.

### Statistical Analysis

PRISM 5 (GraphPad Software Inc., San Diego, CA, USA) was used for all statistical analyses. One-way analysis of variance (ANOVA) testing and Dunn’s multiple comparisons post-test were used to compare data for various groups. Two-way ANOVA with Bonferroni post-test was used to study the simultaneous effect of two variables. Statistical significance was set at *p* < 0.05.

## Results

### Vitamin D Alleviates Pb and Cd-Induced Cytotoxicity to Human Osteoblasts

**Fig. 1:**
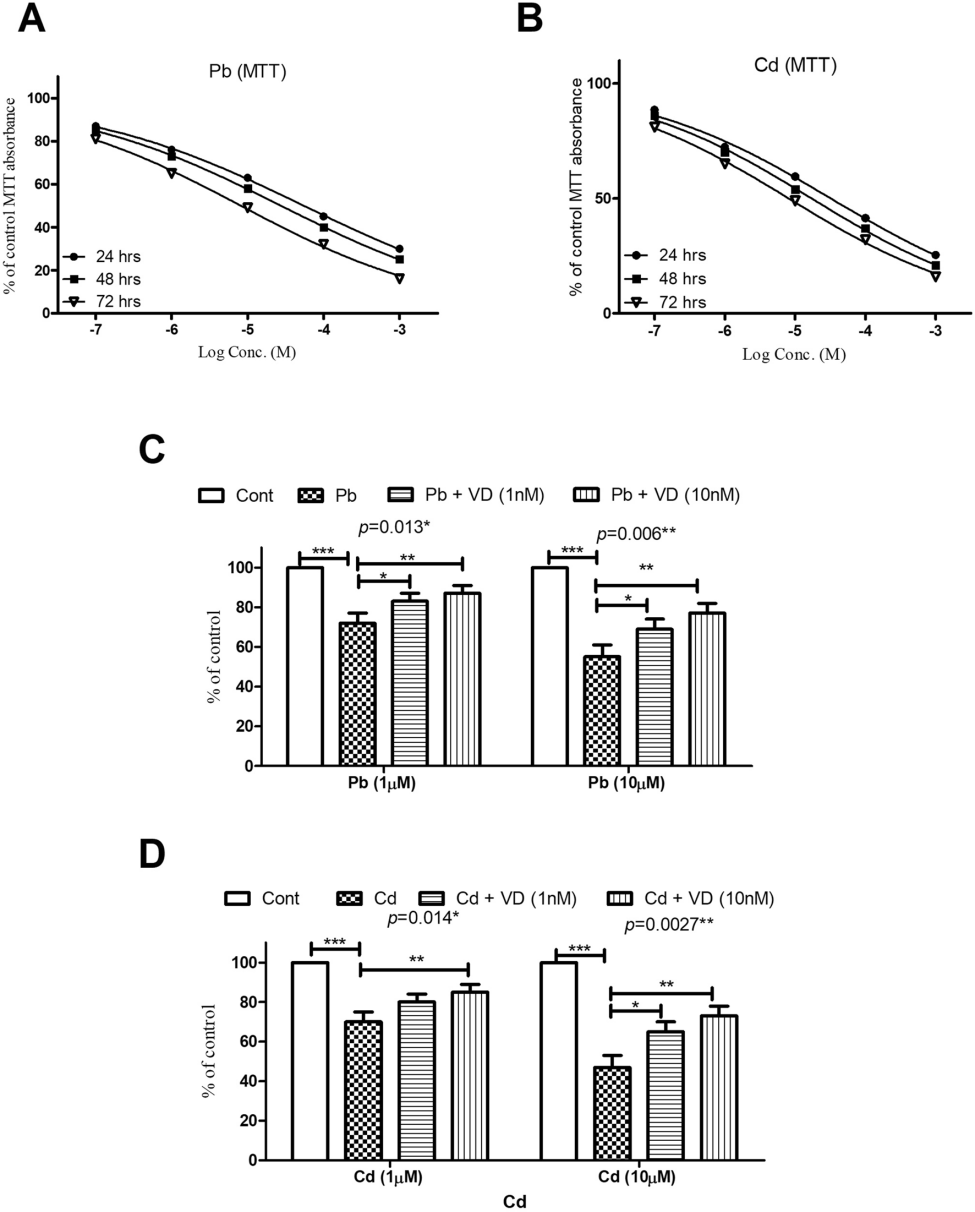
MTT data of the cytotoxic effect of lead (Pb) and cadmium (Cd) (0.1, 1, 10, 100, and 1000 µM) on cultured human osteoblasts at 24, 48, and 72 h post-exposure (**A, B**). **C, D** The protective effect of VD (1 and 10 nM) to alleviate the cytotoxic effects of Pb and Cd on human osteoblasts 72 h post-exposure. Both metals were cytotoxic to the cultured cells in a concentration- and exposure-duration-dependent manner. Cd was more cytotoxic with lower estimated EC50s in the tested time points. Vitamin D significantly alleviated both metals’ cytotoxic effects

Human osteoblasts were exposed to Pb or Cd at concentrations of either 0.1 µM or 1 mM. Cytotoxicity level was quantified after 24, 48, and 72 h using MTT (Fig. 1A, B). Exposure of osteoblasts to Pb and Cd for 48 h was significantly cytotoxic at 0.1 µM (*p* < 0.0001 for both metals). However, Cd exposure was more cytotoxic than Pb with lower IC_50_s at all tested time points with estimated EC50s of 32 (95% confidence interval (CI); 25–36), 17 (95% CI; 12–22), and 8 (95% CI; 4–12) µM 24, 48, and 72 h, respectively. The estimated EC50s for Pb were 55 (95% CI; 49–58), 27 (95% CI; 21–32), and 12 (95% CI; 8–17) µM at 24, 48, and 72 h post-treatment, respectively. Two-way ANOVA showed that Cd and Pb were cytotoxic to osteoblasts in a concentration- and duration-dependent manner and that concentration and exposure duration were responsible for about 35.6 and 53.1% of the variation for Pd and 31.2 and 51.6 for Pd, respectively.

#### Vitamin D Alleviates Pb- and Cd-Induced Disruption in Osteoblast Bioenergetics

**Fig. 2:**
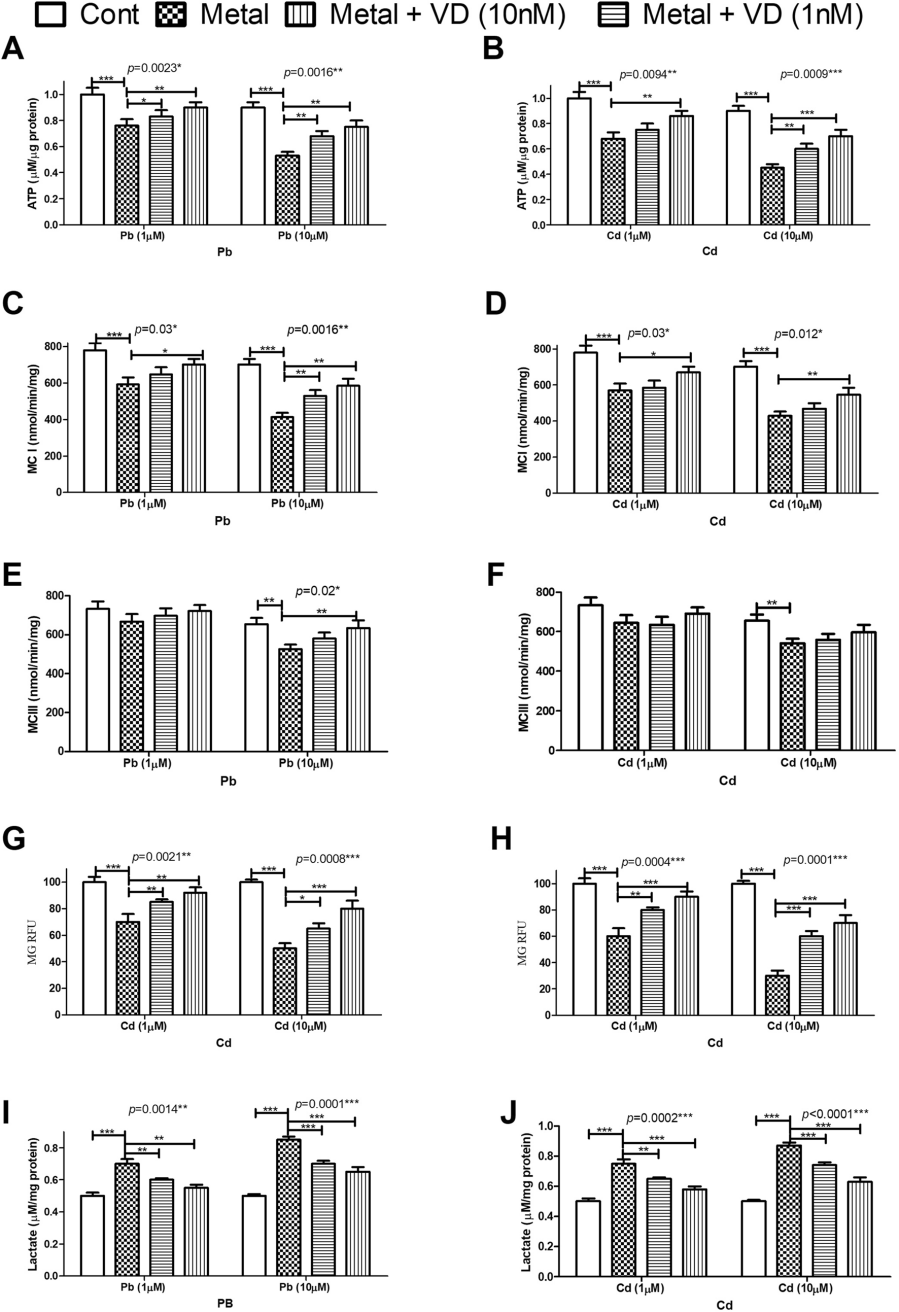
The effect of lead (Pb) and cadmium (Cd) at concentrations of 1 and 10 µM on the cellular bioenergetics of the cultured human osteoblasts and the protective effect of vitamin D (VD) at 1 and 10 nM concentrations. The cells were treated with each metal differently and VD for 72 h. ATP (**A, B**), mitochondrial complex I (MCI) (**C, D**), mitochondrial complex III (MCIII) (**E, F**), mitochondrial membrane potential (MMP) (**G, H**), and lactate production assays (**I, J**) were conducted. Both metals significantly inhibited the treated cells’ bioenergetics in a concentration-dependent manner. Cd showed higher inhibitory effects in all assays. Vitamin D led to significant improvements in the metal-treated cells’ ATP production, higher activity of MMP, MCI, and MCIII, and significantly decreased lactate production. Significance was evaluated by one-way ANOVA with Dunnett’s post-test comparing the outcomes in the presence and absence of VD. ^*^p-value < 0.05, ^**^p-value < 0.01, and^***^p-value < 0.0001

Exposure of osteoblasts to Cd or Pb at concentrations of 1 and 10 µM in the absence or presence of VD (1 or 10 nM) for 72 h showed that Pb significantly decreased intracellular ATP levels to 68% and 46% of the untreated control cells at concentrations of 1 and 10 µM, respectively. Cd was found to significantly reduce intracellular ATP levels to 76% and 53% at concentrations of 1 and 10 µM, respectively. Co-treatment with vitamin D in both tested concentrations alleviated the decreased ATP levels in both metals (*p* < 0.05) (Fig. 2A, B). Since the production of ATP is governed by oxidative phosphorylation linked to the mitochondrial electron transport system, we evaluated membrane potential and mitochondrial complex I and III activity. Both metals were found to significantly decrease the MCI activity in both tested concentrations, while MCIII was only inhibited significantly by both metals at 10 µM. Vitamin D significantly counteracted the effects of both metals on MCI at a concentration of 10 nM. For complex III, VD significantly reduced the effect of Pb at 10 µM concentration with no effect on Cd-induced MCIII inhibition (Fig. 2C**–**F).

Pb and Cd both significantly reduced MMP levels 72 h post-exposure (Fig. 2G, H). At 10 µM concentration, MMP was decreased to 53% for PB and 34% for Cd. Both HMs induced a statistically significant rise in lactate production: At 10 µM concentration, production was around 135% of control levels for Pb and 139% for Cd. Vitamin D was also found to significantly counteract the effect of metals on lactate release (Fig. 2I, J).

#### Vitamin D Alleviates Pb and Cd-Induced Oxidative Damage in Osteoblasts

**Fig. 3:**
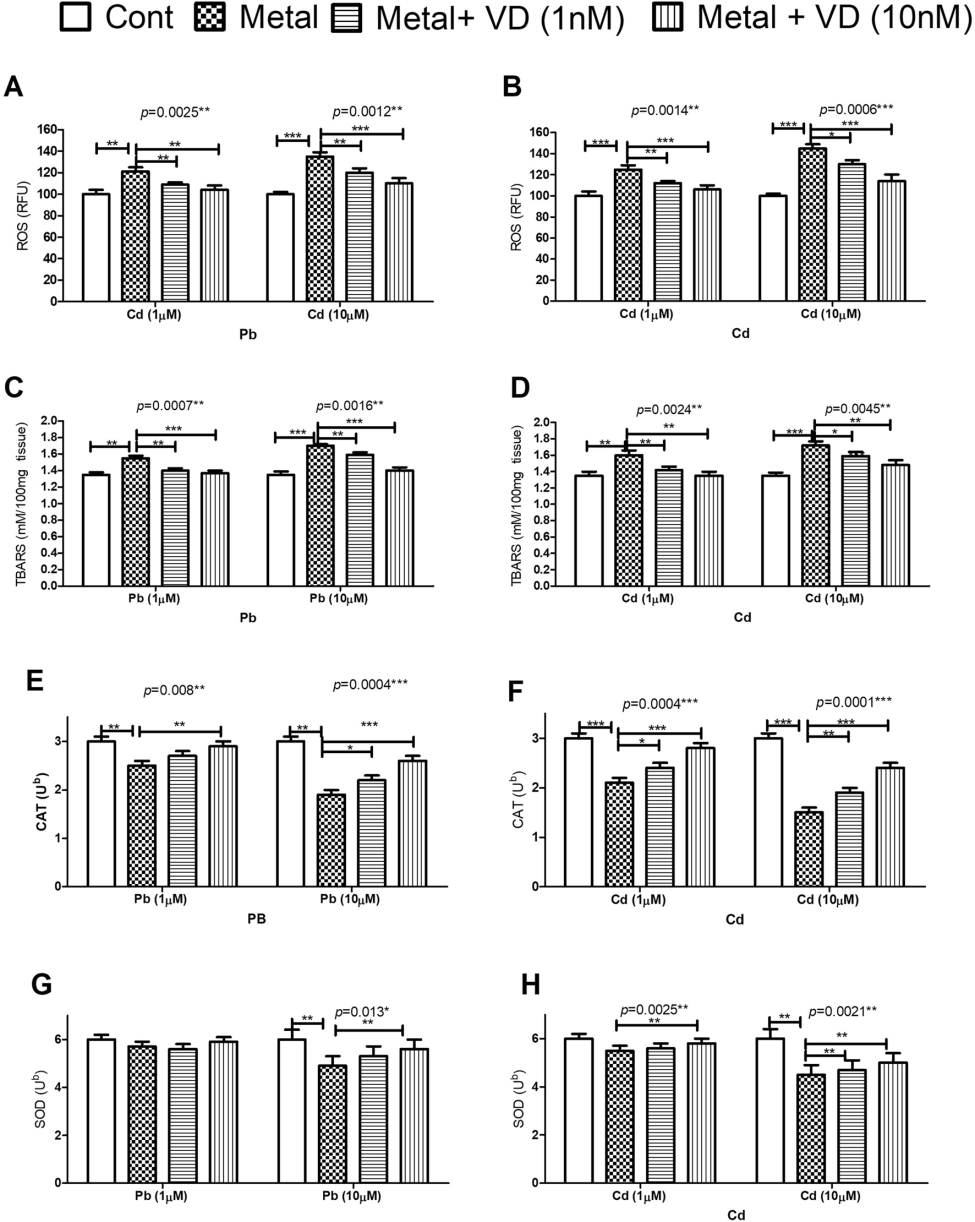
The effect of lead (Pb) and cadmium (Cd) at concentrations of 1 and 10 µM on the redox status of cultured human osteoblasts and the protective effect of Vitamin D (VD) at 1 and 10 nM concentrations. Cells were treated with each metal differently and VD for 72 h. Reactive oxygen species (ROS) (**A, B**), lipid peroxidation thiobarbituric acid (TBARS) product (**C, D**), antioxidant enzyme catalase (CAT) activities (**E, F**), and antioxidant enzyme superoxide dismutase (SOD) activities (**G, H**) were measured. Both metals significantly increased ROS and TBARS production, with a significant decrease in CAT and SOD antioxidant activities in a concentration-dependent pattern. Vitamin D significantly counteracted metal-induced oxidative damage and alleviated the inhibitory effect of the metals on CAT and SOD to varying extents. Significance was evaluated by one-way ANOVA with Dunnett’s post-test comparing the outcomes in the presence or absence of VD. ^*^p-value < 0.05, ^**^p-value < 0.01, and^***^p-value < 0.0001

ROS levels were significantly increased in osteoblasts exposed to Pb and Cd at concentrations 1 or 10 µM in the presence or absence of VD at concentrations 1 and 10 nM for 72 h. Pb and Cd raised ROS levels in treated cells by 132% and 146%, respectively, at a concentration of 10 µM. Co-treatment with vitamin D significantly reduced ROS levels in both Pb and Cd, illustrating the protective effect of VD (Fig. 3A, B). Regarding lipid peroxidation, Pb and Cd both significantly increased TBARS levels, and this effect was significantly decreased by VD co-treatment at 1 and 10 nM (*p* < 0.05) (Fig. 3E). Pb significantly reduced CAT activity to 83.3% of controls at 1 µM concentration and 63.5% at 10 µM. Cd significantly reduced CAT activity to 71.2% of controls at 1 µM and 52.5% at 10 µM. Vitamin D significantly increased CAT activity, suggesting an alleviation effect based on concentration. At 10 nM concentration, VD improved the CAT activities by 23% and 31% of controls in samples treated at the 10 µM concentration level for Pb and Cd, respectively. Metals were found to affect SOD activities to a lesser extent than CAT. Vitamin D counteracted the effect of Pb (10 µM) and Cd (1 µM) on SOD activities in both tested concentrations.

#### Vitamin D Alleviates Pb and Cd-Induced Apoptosis of Human Osteoblasts

**Fig. 4:**
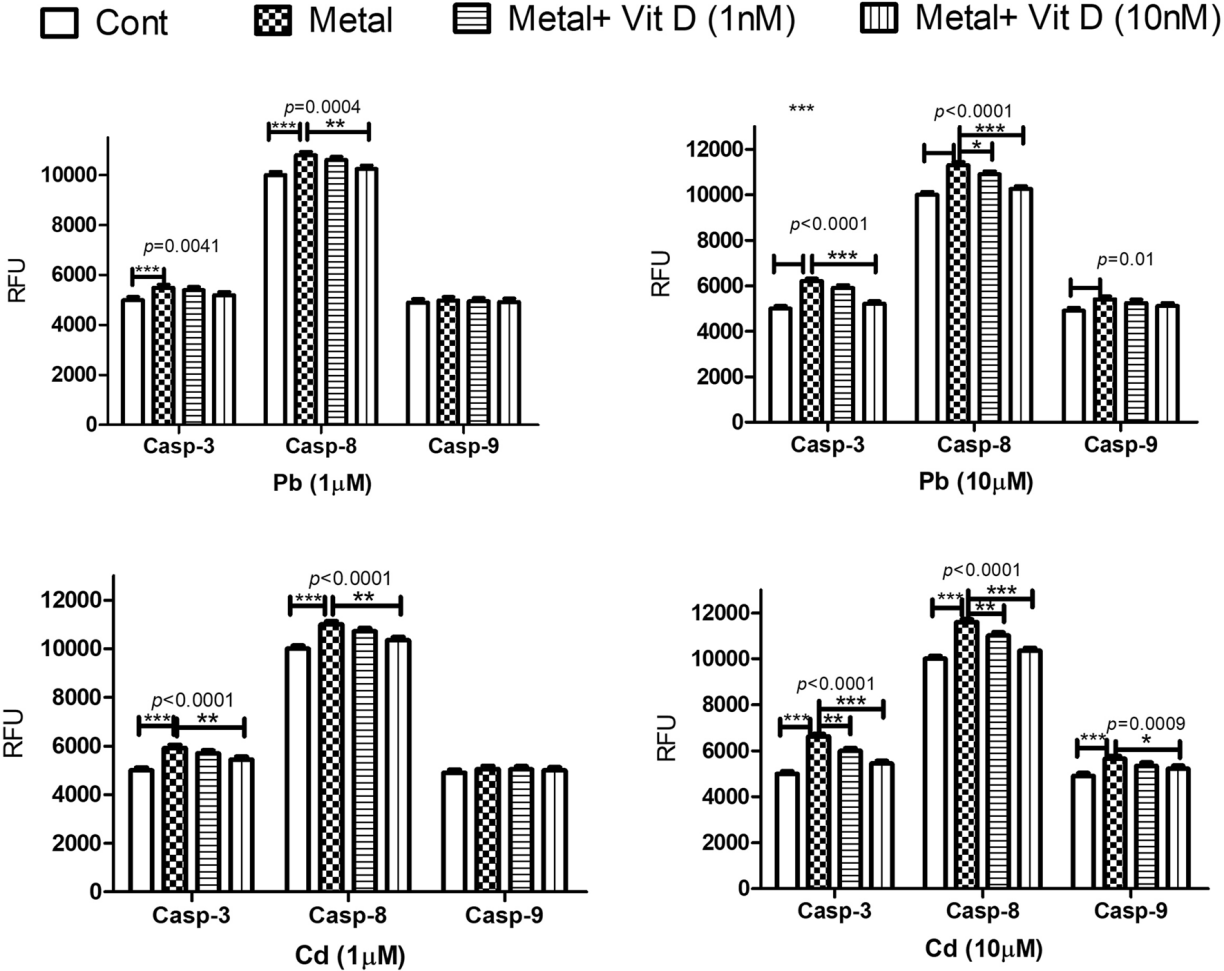
The effect of lead (Pb) and cadmium (Cd) at 1 and 10 µM concentrations, 72 h post-exposure, on caspase-3, -8, and -9 activity in cultured human osteoblasts and the protective effect of Vitamin D (VD) at concentrations of 1 and 10 nM. Both metals significantly inhibited the secretory functions of the treated cells in the three assays in a concentration-dependent manner. Vitamin D significantly counteracted the metal-induced secretory dysfunction of the treated cells. Significance was evaluated by one-way ANOVA with Dunnett’s post-test comparing the outcomes in the presence and absence of VD. ^*^p-value < 0.05, ^**^p-value < 0.01, and ^***^p-value < 0.0001

The effect of metals on human osteoblast apoptotic pathways was evaluated by studying caspase-3 as a marker for common apoptotic pathways, caspase-8 for intrinsic pathways, and caspase-9 for extrinsic pathways. Both Pb and Cd were found to significantly increase the activity of caspase-3 and -8 at concentrations of 1 µM and 10 µM, while caspase-9 was increased only at 10 µM (Fig. 4). Vitamin D at 1 nM significantly decreased the effect of both metals at 10 µM concentrations on caspase-3 and -8.

#### Vitamin D Alleviates the Pb and Cd-Induced Functional Derangement in the Human Osteoblasts

**Fig. 5:**
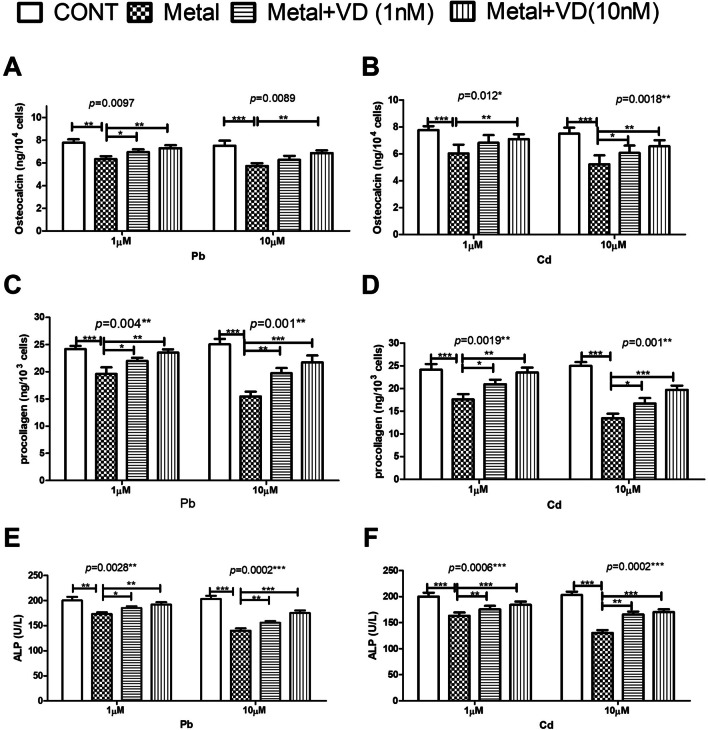
The effect of lead (Pb) and cadmium (Cd) at concentrations of 1 and 10 µM, 72 h post-exposure, on osteocalcin (**A, B**), procollagen type I peptide (**C, D**) and alkaline phosphatase (ALP) (**E, F**) production in cultured human osteoblasts. Both metals significantly inhibited the secretory functions of the treated cells in the three assays in a concentration-dependent manner. Vitamin D significantly counteracted the metal-induced secretory dysfunction of the treated cells. Significance was evaluated by one-way ANOVA with Dunnett’s post-test comparing the outcomes in the presence and absence of VD. ^*^p-value < 0.05, ^**^p-value < 0.01 and.^***^p-vale < 0.0001

Functional assays revealed that at 72 h post-exposure, both metals at both concentrations (1 and 10 µM) significantly decreased osteoblast secretion of osteocalcin (Fig. 5**A**, **B**), procollagen type I peptide (Fig. 5**C**, **D**), and ALP (Fig. 5**E**, **F**) to varying extents based on concentration. Procollagen type I peptide was the most strongly affected at all tested concentrations. Cd produced more inhibitory effects in all assays than Pb at the same concentrations, and VD decreased metal-induced inhibitory effects on osteoblast secretory functions in a concentration-dependent manner.

## Discussion

We tested the protective effect of VD against the cytotoxic impacts of two commonly encountered heavy metals, lead (Pb), and cadmium (Cd), on human osteoblasts. Various assays covered cytotoxic mechanisms, including bioenergetics, oxidative damage, and apoptosis. Although bone comprises osteoblasts, osteocytes, osteoclasts, and bone lining cells, we used a homogenous population of human osteoblasts to distinguish effects exclusive to this particular cell type. Using a human osteoblast cell line instead of primary cells prevented species-specific reactions, ensured phenotypic consistency, and provided results consistent with primary cell studies [21]. For the applied assays in this study, PB and Cd were tested at a concentration of 10 µM within the MTT assay estimated EC50s with a 95% confidence interval (CI) for both metals 72 h post-exposure. Additionally, both metals were tested at a 1 µM concentration, one-tenth of their estimated EC50s, to evaluate the effects of lower concentration exposure. Testing both metals at the same concentrations allows for directly comparing their cytotoxic effects. Meanwhile, VD was tested at 1 nM and 10 nM concentrations, as previous studies on the same cell line have shown these concentrations to be effective (Tong et al., 2023). Also, these concentrations have been proven to improve the viability of human osteoblast passage in our lab (unpublished data).

According to the World Health Organization (2018), there is no safe level of lead exposure. It affects multiple body systems, including the skeletal system. Clinical symptoms of lead poisoning, such as kidney dysfunction, begin to appear at blood lead levels (BLL) of 5–10 µg/dL (0.24–0.48 µM). More severe health effects are observed at higher BLLs, including encephalopathy at 100–120 µg/dL (≈6 µM), neuropathy at > 80 µg/dL (≈4 µM), and cognitive impairment at 40–79 µg/dL [22]. To accurately reflect real-world exposure, this study examined a wide range of lead concentrations, from 0.1–1 µM (symptomatic levels) to > 10 µM (extreme poisoning), levels that are comparable with historical and current BLLs [23].

Cadmium excretion in the urine, often measured over 24 h, is a common indicator of cadmium accumulation in the body. Blood cadmium levels as high as 0.145 µmol/L and BLLs of 1–3.7 µmol/L have been recorded in workers exposed to Pb and Cd, such as those in smelting jobs [24]. Using a similar concentration range for Pb and Cd, we could directly compare their toxicities and determine whether similar mechanisms of toxicity emerged at specific exposure doses.

In vitro, chronic heavy metal exposure at higher doses often represents cumulative exposure over a shorter study period. Concentrations exceeding 10 µM are considered overdose levels. The accumulation of lead and cadmium in bone is particularly worrying, since these metals are non-degradable and have slow clearance rates, with lead having a half-life of about 40 days in blood and cadmium around 20–30 years [25].

**Fig. 6:**
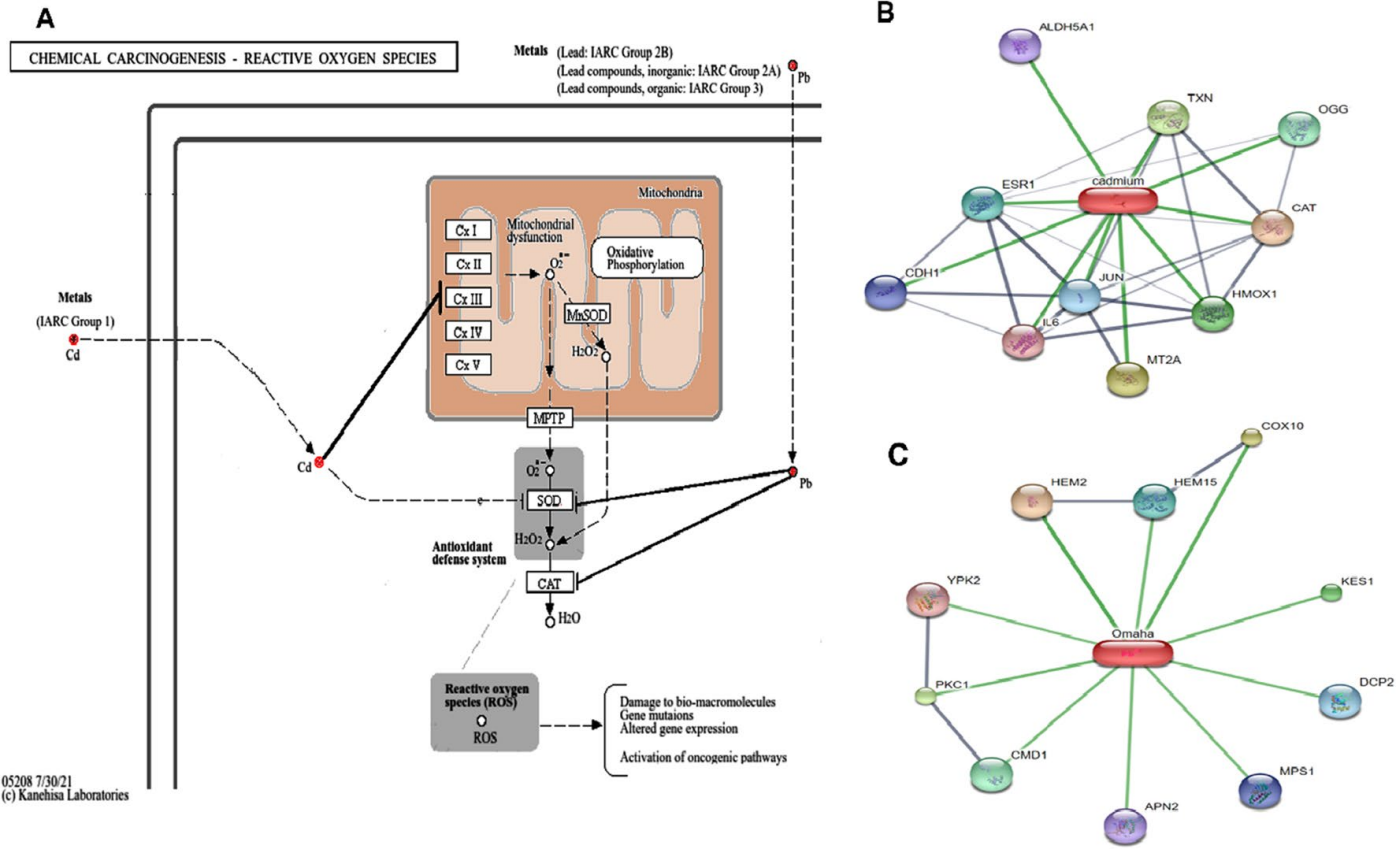
Potential mechanisms of cadmium (Cd) and lead (Pb) cytotoxicity. (**A**) Trace metals have prooxidant and mitochondrial disruption effects that lead to the generation of reactive oxygen species (ROS), depending on their ability to lose electrons. Elevated ROS production is frequently associated with DNA damage and can trigger various intracellular signaling pathways, including apoptotic pathways (Adopted with permission from the Kyoto Encyclopedia of Genes and Genomes (KEGG) (https://www.kegg.jp/pathway/map05208) [36]. (**B**) Predicted protein interaction network of cadmium: CAT: catalase; MT2A: metallothionein 2A; TXN thioredoxin; HMOX1 heme oxygenase; OGG1 8-oxoguanine DNA glycosylase; ESR1: estrogen receptor 1; JUN: jun proto-oncogene; CDH1: cadherin 1; ALDH5A1 aldehyde dehydrogenase 5 family member A1; and IL6: interleukin 6. (**C**) Predicted protein interaction network of lead: PKC1 protein serine/threonine kinase; KES1 (member of the oxysterol binding protein); CMD1: calmodulin; DCP2 (catalytic subunit of the Dcp1p-Dcp2p decapping enzyme complex); MPS1 (dual-specificity kinase required for spindle pole body duplication and spindle checkpoint); APN2 Class II (a basic endonuclease involved in DNA damage repair); YPK2: protein kinase; HEM2: heme A-farnesyltransferase; and HEM15: ferrochelatase. These interactions explain some of the oxidative stress-, apoptosis-, and mitochondrial disruption-related effects of these metals. Graphical representations of the potential protein partners of Cd and Pb were identified from the STITCH database (http://stitch.embl.de/) [37]

Our study demonstrates that Cd and Pb significantly impair the viability of human osteoblasts in a time- and concentration-dependent manner, with Cd exhibiting a more pronounced cytotoxic effect compared to Pb. The half-maximal effective concentration (EC50) values for Cd and Pb were found to be 8 µM and 12 µM, respectively, after 72 h of exposure. This differential toxicity suggests that Cd may be more potent in disrupting cellular functions, which could be attributable to its higher affinity for thiol groups in proteins and its ability to mimic essential divalent cations such as calcium and zinc, thereby interfering with various cellular processes [26]. Vitamin D co-treatment significantly increased cell viability and reduced metal-induced cytotoxicity. To understand the mechanism of VD’s cytoprotective effects, we investigated its impact on various mechanisms of metal cytotoxicity (Fig. 6). VD mitigated oxidative stress, apoptosis, and mitochondrial disruption induced by heavy metals. Over 200 genes related to cellular differentiation, proliferation, apoptosis, angiogenesis, and inflammation are influenced by VD [27]. It is known to upregulate the expression of antioxidant enzymes such as catalase and superoxide dismutase [28], which were observed to be reduced upon exposure to Cd and Pb. This upregulation likely contributes to the observed decrease in ROS and lipid peroxidation, counteracting the oxidative stress induced by heavy metals.

Further mechanistic insights can be drawn from the bioenergetics assays, where both Cd and Pb were found to inhibit mitochondrial complex I and III activities, leading to a drop in ATP levels and mitochondrial membrane potential. VD co-treatment restored these parameters toward normal levels, suggesting that VD might preserve mitochondrial integrity and function. One plausible mechanism is that VD enhances the activity of mitochondrial biogenesis pathways or stabilization of mitochondrial membranes, which supports cellular ATP production and overall bioenergetic balance [29]. Using microarray analysis, the latter investigators reported evidence suggesting that vitamin D’s impact on muscle mitochondria is mediated through a nuclear mechanism that influences the expression of key regulators of mitochondrial biogenesis and function. They found that mitochondrial genes were most regulated by VD, suggesting the possibility of a direct mitochondrial VD pathway that can rapidly enhance mitochondrial oxidative activity.

The literature supports VD's role in enhancing bioenergetics. Ryan et al. [30] reported increased mitochondrial oxygen consumption and volume in human skeletal muscle cells treated with VD. Vitamin D also affects calcium and phosphorus uptake along with gene expression, proliferation, and differentiation in skeletal muscle cells [31, 32]. Healthy mitochondria produce ATP, essential for cellular function, while damaged mitochondria generate harmful ROS [33]. Oxidative stress regulates mitochondrial fission, and excessive fission can lead to mitochondrial dysfunction and cellular damage [34, 35].

VD significantly reduced oxidative damage in osteoblasts. Studies have shown VD’s anti-oxidative properties across different tissues, including the liver [38], kidneys [39], heart [40], brain [41], and adipose tissue [42]. Vitamin D has also been shown to counteract mitochondrial stress and ROS production in various tissues [43–46]. The current study found that VD treatment increased the enzymatic activities of CAT and SOD in osteoblasts, consistent with other research showing that VD upregulates antioxidant gene expression under stress [44, 47].

In addition to its effects on oxidative stress and bioenergetics, VD also played a substantial role in modulating apoptosis. The activation of caspases -3, -8, and -9 in response to Cd and Pb indicates the involvement of both intrinsic and extrinsic apoptotic pathways. VD’s ability to prevent the activation of these caspases suggests an anti-apoptotic role, potentially through the upregulation of anti-apoptotic proteins such as Bcl-2 and inhibition of pro-apoptotic factors [48]. VD may also interfere with signaling pathways involved in apoptosis, such as the NF-κB pathway [49], thereby providing a survival advantage to the osteoblasts under heavy metal stress. Vitamin D also significantly inhibits apoptosis; in cardiac myocytes, VD reduces mitochondrial apoptosis by decreasing cytochrome c expression [50]. VD reduced superoxide production and apoptosis in endothelial cells, mitigating H_2_O_2_-induced stress in a dose- and time-dependent manner [46].

The cytoprotective mechanisms of VD, bioenergetics, oxidative stress reduction, and apoptosis inhibition are interrelated. Mitochondria are primary sources of ROS during normal physiological processes. Disruption of bioenergetics and inhibition of the electron transport chain lead to increased ROS production [51]. Excessive oxidative stress can damage cellular structures, triggering further mitochondrial disruption and increased ROS generation [52], which, in turn, can activate apoptotic pathways and elevate cytosolic cytochrome c [53]. Excessive oxidative stress can lead to cell death via apoptosis or necrosis [54]. In several apoptosis models, a shift toward a more oxidizing cellular environment precedes caspase activation [55].

The current findings are significant since they illustrate VD’s role in controlling bone diseases characterized by oxidative stress, apoptosis, and mitochondrial dysfunction, such as osteoporosis, osteoarthritis, peri-implantitis, and bone remodeling disorders [56]. The concentration-dependent protective effects of VD observed in our study also highlight the importance of optimal VD levels in mitigating environmental toxicity. Lower concentrations of VD (1 nM) were effective, but higher concentrations (10 nM) provided more robust protection against Cd- and Pb-induced cytotoxicity. This dose–response relationship underscores VD’s potential as a dose-dependent therapeutic agent in counteracting heavy metal toxicity on bone physiology and dynamics.

## Strengths and Limitations of the Study

Although this study (a) evaluated multiple cytotoxic mechanisms, including bioenergetics, oxidative damage, and apoptosis, providing a well-rounded understanding of the effects of heavy metals on osteoblasts; (b) employed a human osteoblast cell line, ensuring that the results are directly applicable to human bone cells, avoiding potential interspecies differences that can arise with animal models; (c) addressed a pressing public health issue, as the findings have direct implications for understanding and mitigating the health risks associated with environmental pollutants; and (d) highlighted the potential therapeutic benefits of vitamin D, a widely available nutrient, to reduce the health impacts of heavy metal exposure; some potential limitations should be considered. (a) The study was conducted in vitro using a human osteoblast cell line, which may not fully replicate the complexity of in vivo bone environments; (b) the use of a single human osteoblast cell line is less ideal than using primary cells derived from multiple donors; (c) the concentration ranges and exposure time of Cd and Pb may not cover all possible exposure levels and duration that might occur in real-world scenarios; (d) in-depth exploration of other potential cellular mechanisms and pathways/markers (including other apoptotic biomarkers) was beyond the scope of the current study; and (e) the study did not include in vivo experiments to corroborate the in vitro findings.

## Future Perspectives

Future studies should seek to validate the in vitro findings in animal models or clinical trials. In vivo studies will help to confirm the protective effects of vitamin D against heavy metal-induced cytotoxicity in a more complex biological context. Further research should also investigate the specific molecular pathways through which vitamin D exerts its protective effects. Expanding the scope to include other heavy metals such as mercury (Hg), arsenic (As), and chromium (Cr) could provide a more comprehensive understanding of how vitamin D might mitigate a wider spectrum of heavy metal toxicities. Investigating a broader range of vitamin D doses and extended exposure periods will help to determine optimal dosing strategies and assess the potential benefits and risks of prolonged vitamin D supplementation.

Exploring the synergistic effects of vitamin D and other antioxidants/protective agents could lead to developing more effective intervention strategies against heavy metal toxicity. Research into the genetic and epigenetic factors modulating the response to vitamin D and heavy metal exposure could identify susceptible populations for personalized intervention strategies. Lastly, encouraging collaboration between fields such as toxicology, nutrition, environmental science, and public health will enrich our understanding and application of vitamin D’s protective effects against heavy metal toxicity.

## Data Availability

Data will be available upon request from the corresponding author.

## Abbreviations

As: Arsenic
ALP: Alkaline phosphatase
Bax: Bcl-2 associated X-protein
BMD: Bone mineral density
CAT: Catalase
Cd: Cadmium
H_2_O_2_: Hydrogen peroxide
hBMSCs: Human bone marrow stromal cells
HMs: Heavy metals
Hg: Mercury
MMP: Mitochondrial membrane potential
NPMSCs: Nucleus pulposus-derived mesenchymal stem cells
OCN: Osteocalcin
Pb: Lead
PIP: Procollagen type I C-Peptide
SOD: Superoxide dismutase
TBARS: Thiobarbituric acid reactive substances
VD: Vitamin D
VDR: Vitamin D receptor
